# Frequency Maps of FTIR Images for Phenotyping Molecular Heterogeneity of the Primary Tumor and Its Metastases

**DOI:** 10.1021/acs.analchem.5c05310

**Published:** 2026-06-08

**Authors:** Karolina Chrabaszcz, Kamilla Malek

**Affiliations:** † Institute of Nuclear Physics, Polish Academy of Sciences, Radzikowskiego 152, 31-342 Krakow, Poland; ‡ Jagiellonian University in Krakow, Faculty of Chemistry, Gronostajowa 2, 30-387 Krakow, Poland

## Abstract

Cancer cells within a single tumor exhibit significant morphological, genetic, and metabolic variability, posing challenges for identifying and treating carcinoma by selecting suitable biomarkers. This complexity necessitates multiparametric characterization, such as next-generation sequencing and proteomics, which complicates their practical clinical management and the implementation of precision medicine. Here, we propose a new analytical approach for phenotyping cancer heterogeneity using hyperspectral IR images of tumors and maps of frequency shifts. For this purpose, we focus on the molecular drivers underlying the features of cancer cells and their metabolism, which are unequivocally identified in FTIR spectra, including the DNA and protein conformational landscape, carbohydrate metabolism, and extracellular matrix digestion. Since intratumor heterogeneity is a dynamic process and depends on the microenvironment and expanding tumor mass, we tested our method on the murine model of pulmonary metastasis of mammary gland carcinoma, delivering primary and secondary tumors. The frequency maps captured inter- and intratumor diversity, providing molecular information which can be linked to metastatic evolution.

## Graphical abstract

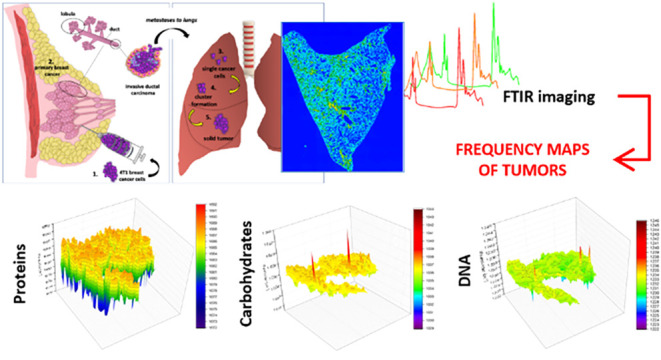

Nearly all breast cancer-related deaths result from metastasis to distal organs or recurrence at the site of the primary tumor.^1^ Another well-known fact is the extensive genetic and epigenetic variability in the growth of primary tumors and metastases. Primarily, a small population of the neoplastic cells in primary invasive cancer undergoes genetic aberrations and drives the tumor progression, and this phenomenon is particular to breast cancer.^2^ Several reports have shown cancer cells’ phenotypic and genetic diversity by identifying and quantifying proteins and genes using immunohistochemistry, mass spectrometry-based proteomics, fluorescence in situ hybridization (iFISH), Next Generation Sequencing (NGS), comparative genomic hybridization, and migratory ability.^3,4^ Next, these outcomes were correlated with histopathological features of tumors and metastatic ability to predict the likelihood of metastasis in patients with clinically diagnosed cancer, as well as to prevent therapeutic resistance. Several clinical, in vitro, and ex vivo studies have proven that cancer cell heterogeneity exists both between and within individual tumors, and is responsible for the ability to migrate to distant organs.^5,6^ The critical issue in the early recognition of the metastatic process is the complexity of biomarkers of highly migratory and metastatic populations. Moreover, given the tremendous challenge of the investigations needed to characterize biomarkers that explain tumor heterogeneity, there remains a need for new methods that introduce novel concepts for these diversity measures and at least provide effective prescreening to enable further specific examinations, such as proteomics, metabolomics, and genotyping in animal and clinical studies.

Label-free molecular imaging using Fourier transform infrared (FTIR) spectroscopy has already been offered as a powerful and automatic method for cancer detection, identification of its malignancy, and carcinoma-associated processes, particularly when combined with machine learning.^7–10^ Vibrations of chemical species induced by IR light absorption reveal a unique signature of cells in a tissue encoded in an IR image, even though their gross biochemical composition is similar. By using clustering methods to group similar features, each pixel in the image is assigned to a specific tissue cell. At the same time, its spectral profile represents the biochemical composition of that cell. Our previous reports have discussed the utility of FTIR imaging for visualizing early pulmonary metastases, classifying cancer subtypes, predicting metastasis, and detecting biochemical changes in the surrounding microenvironment, including extracellular matrix remodeling, ordering of fibrous proteins, or metabolic reprogramming that occur during the metastasis process.^11–14^ However, there is still a niche for deeper insights into the hyperspectral data sets to reveal characteristics of the metastatic cascade.

To address the inter- and intratumor heterogeneity of cancer cells, we investigated mammary carcinoma 4T1 cells orthotopically injected into BALB (Bagg Albino) mice to model aggressive human breast cancer subtypes, such as basal, Human Epidermal growth factor Receptor 2 (HER2), and claudin-low, with well-established pulmonary metastasis. This animal model is among the most reliable for mimicking human breast cancer.^14^ We chose this animal model to investigate cancer growth and metastasis under control conditions, since access to well-defined patient biopsies containing various metastatic tumor sizes of different locations in the lung is limited. Primary tumors and 4T1 cells from the *in vitro* system were first examined. Then we selected pulmonary lesions grown *in vivo* representing extravasation, metastatic colonization, and epithelial-to-mesenchymal transition (EMT), cf. Figure 1. Using an unsupervised segmentation method of IR images, breast cancer cells were spectrally extracted from the tissue matrix, and their inherent biochemical content was assessed to indicate the most substantial differences in the fingerprint region of the FTIR spectrum between the phases of the cancer disease. Finally, we captured chemical variation within the spatial organization of lesions by constructing three-dimensional (3D) frequency maps of proteins, nucleic acids, and carbohydrates, revealing the inter- and intraheterogeneity in cell phenotypes of cancer lesions. To the best of our knowledge, this is the first report in the literature to propose this approach in spectral histopathology, which could be applied in subsequent animal studies to examine metastasis mechanisms or new treatment effects, and next, possibly translated to clinical samples.

**Figure 1:**
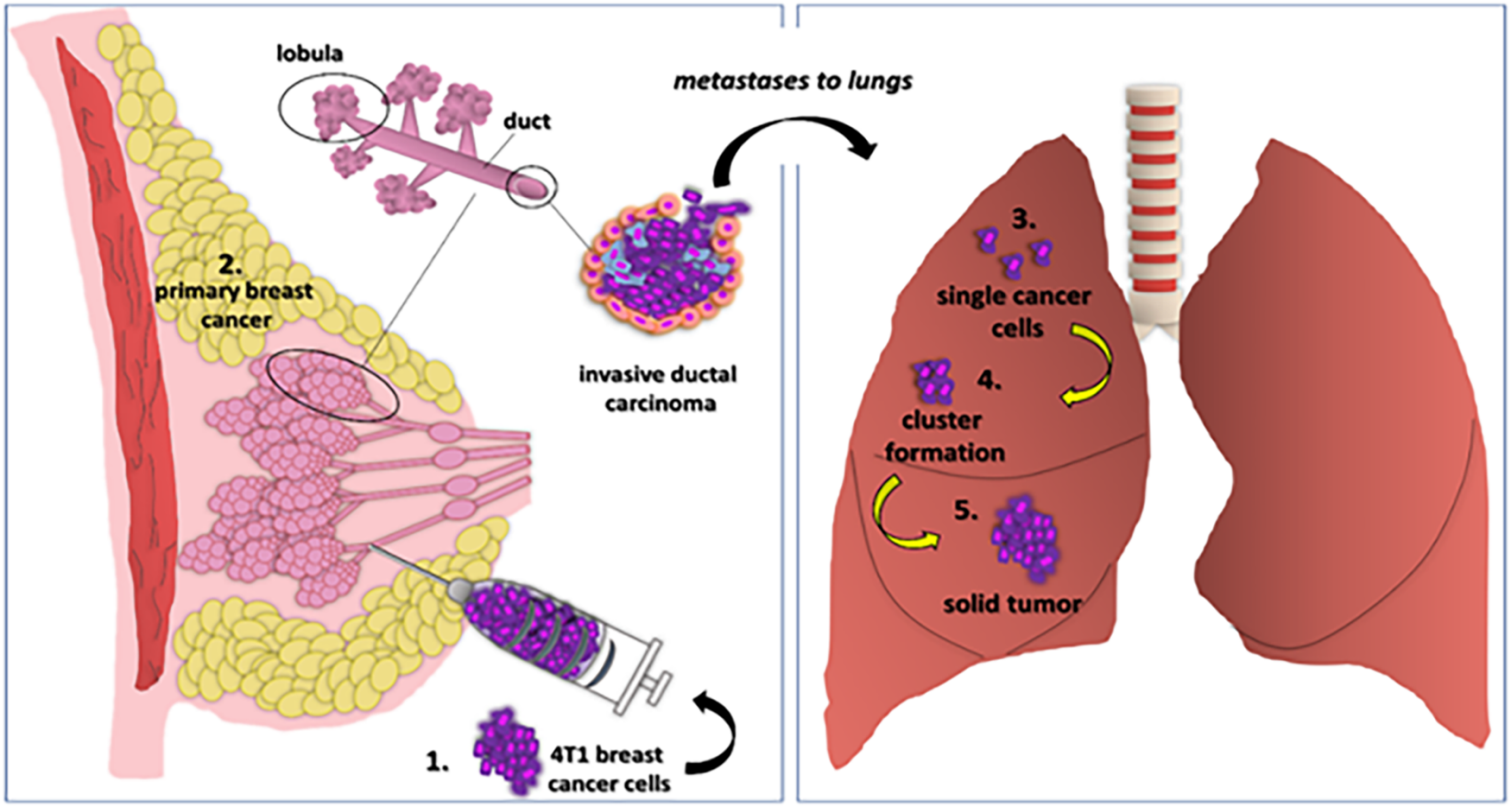
A schematic showing a route of 4T1 cells administered to the mammary gland of BALB/cJRj mice and phases of their growth in the primary tumor and lung.

## Materials and Methods

### Cell Culture, Animal Model, and Histological Staining

The mouse 4T1 cells were obtained from the American Type Culture Collection (ATCC). Cells were cultured in RPMI 1640-Glutamax medium (Sigma-Aldrich, Poland) supplemented with 10% fetal bovine serum (Gibco, Thermo Fisher Scientific, Poland), 1.0 mM sodium pyruvate (Sigma-Aldrich, Poland), and antibiotic antimycotic solution (100 units/mL penicillin and 100 μg/mL streptomycin, 25 μg/mL amphotericin B) (Sigma-Aldrich, Poland). Cells were cultured at 37 °C in a humidified atmosphere containing 5% CO_2_. Only 4T1 cells at the second passage were used for inoculations. Before the transplantations, 4T1 cells were detached using an Accutase solution (Sigma-Aldrich, Poland), centrifuged (300*g*, 4 °C, 5 min), counted, suspended in Hank’s Balanced Salt Solution (HBSS; IIET, Poland) at the appropriate concentration, and inoculated into the mammary gland of female BALB/C mice. Routine tests for Mycoplasma contamination (MycoAlert Mycoplasma Detection Kit; Lonza) were performed on all cell cultures. For IR imaging, 4T1 cells were grown on calcium fluoride slides (CaF_2_) and then fixed with 4% glutaraldehyde (three replicates). After 24 h, they were fixed for 4 min in 4% glutaraldehyde and stored in isotonic phosphate buffer (pH 7) at 4 °C until data acquisition. Primary tumors were obtained from inbred mouse strains BALB/cAnNCrl (*n* = 4) within 5 weeks after orthotopic inoculation with viable 4T1 cells. After mice euthanasia, primary tumors were excised and fixed using (1) optimal cutting temperature medium (OCT). The primary breast cancer tissue was washed with a saline solution and then a 1:1 mixture of saline and OCT. The tumor was embedded in OCT and frozen at −80 °C. Seven μm-thick cross sections were cut in an electromechanical cryostat (Leica CM 1950) and placed on a CaF_2_ window. After fixation in 4% buffered formalin solution, tissue cross sections were washed out with distilled water and air-dried for 20 min before IR imaging. The other primary tumor was fixed using (2) a formalin-fixed paraffin embedding method (FFPE); the tissue was washed with saline and fixed with a 4% buffered formalin solution for 48 h and then embedded in paraffin. Tissue blocks were then cut (7 μm) using an Accu-Cut SRM 200 Rotary microtome. Before IR imaging, the tissue cross sections were mounted on CaF_2_ windows and dewaxed (ethanol 96%, xylene). Lung samples were from inbred mouse strains BALB/cAnNCrl (*n* = 3) after 3, 4, and 5 weeks after orthotopic inoculation with the viable 4T1 tumor cells. Isolated lungs were washed in saline, fixed, and sectioned using the FFPE method as above. The pure paraffin and OCT, as well as tissue spectra after the deparaffinization and water rinsing, were presented in Figure S1 in Supporting Information (SI).

After IR imaging, tissue cross sections were stained with hematoxylin and eosin (H&E) for the histopathological examination. The examination and photographic documentation were performed using an Olympus BX53F white-light microscope equipped with a DP74 digital camera.

All experimental procedures involving animals have been carried out according to the Polish Research Council’s Guide for the Care and Use of Laboratory Animals under the consent issued by the Second Local Ethical Committee on Animal Testing, Institute of Pharmacology in Krakow, Poland (Permit Nos: 61/2020, 167/2020, 228/2020, and 66/2021).

### IR Spectroscopy Imaging and Data Analysis

#### Measurements

A combination of an Agilent 670-IR FT-IR spectrometer and a 620-IR microscope, coupled with a focal plane array (FPA) detector, was employed. The detector consists of a 16,384-pixel matrix arranged in a 128 × 128 grid. IR images were acquired in transmission mode. A Cassegrain objective with a numerical aperture (NA) of 0.62 and a projected pixel size of 5.5 μm was used (Standard mode, SD). An HD IR imaging mode, developed by Agilent Technologies, enables enhanced spatial resolution optics using the Cassegrain 15× objective lens. FT-IR spectra of tissue cross sections were recorded by coadding 32 (SD mode) and 128 scans (HD mode) in the range of 3800–900 cm^–1^ with a spectral resolution of 4 cm^–1^.

#### Preprocessing of Spectral Data Sets

Preprocessing and chemometric analysis were performed using CytoSpec (v.2.00.01),^15^ MATLAB (v.R2015a), and Origin (v.2018b, OriginLab) software. If necessary, a water vapor correction was applied. A quality test was used to establish a threshold and eliminate signals with absorbance values below 0.2 or above 1.2. This operation was performed in the region between 1620 and 1680 cm^–1^. To remove spectral noise, Principal Component Analysis-based noise reduction with 15 principal components was executed.

#### Cluster Analysis

First, the second derivative IR spectra were calculated with 13 smoothing points using the Savitzky–Golay method. Second-derivative spectra were used to improve the detection of band positions in regions where IR bands partially overlap with neighboring signals or appear as shoulders on broader bands, thereby enhancing spectral resolution and reducing baseline contributions. Before conducting further cluster analysis, all spectra were vector normalized in the 914–1770 cm^–1^ region to avoid differentiation due to sample thickness. Using second derivative FT-IR spectra, unsupervised hierarchical cluster analysis (UHCA) was executed in the 970–1770 cm^–1^ region. Spectral distances were computed as D-values, and individual clusters were extracted according to Ward’s algorithm. The number of classes was selected to maximize differentiation of FT-IR spectra and to replicate the tissue’s morphological structure as observed by H&E staining according to our previously established protocols.^12,13^ Resonant Mie Scattering (RMieS) correction using seven principal components was performed on all normal FT-IR spectra.^16^ The second derivative FT-IR spectra extracted from the calculated UHCA classes were displayed using the Origin software. The comparative analysis was performed on the vector-normalized spectra in the entire region.

#### Frequency Maps

Frequency maps were generated using the frequency imaging function in CytoSpec to visualize spatial variations in band positions across the tumor regions in IR images separated by UHCA. The frequency map shows band positions at each pixel, represented as color-coded values plotted as a function of spatial coordinates (Origin). The maps were computed in the spectral regions attributed to DNA (1246–1222 cm^–1^), carbohydrates (1044–1029 cm^–1^), protein turns and intermolecular aggregates (1692–1672 cm^–1^), collagen hydroxyproline (1170–1140 cm^–1^), and elastin (1074–1048 cm^–1^). We used absorbance and second-derivative FTIR spectra (13 smoothing points, Savitzky-Golay algorithm) preprocessed as described above. A cubic spline algorithm with factors of 8 and 16 was applied to achieve precise band searching and localization and to increase the density of spectral points within the selected interval. The algorithm subsequently identifies the x-position (wavenumber) of the maximum/minimum within the specified spectral region for each spectrum in the hyperspectral data set. Since the spectra were recorded with a spectral resolution of 4 cm^–1^ and a spectral sampling interval of approximately 1.9 cm^–1^ (1000.88–3600.50 cm^–1^ over 1349 points), the interpolation factors of 16 and 8 result in an interpolated numerical spacing of approximately 0.12 and 0.24 cm^–1^ (1.9 cm^–1^/16; 1.9 cm^–1^/8), respectively. This interpolation improves the numerical precision in locating the derivative minimum but does not increase the instrument’s intrinsic spectral resolution. The frequency distributions were visualized using histograms with a 1 cm^–1^ bin width using Origin to show systematic shifts in band-position distributions while avoiding oversampling relative to the nominal spectral resolution. Pixels with abnormal spectral baselines or insufficient signal intensity were excluded from the analysis. A summary of the algorithm is provided as pseudocode in Note S1 in SI.

#### Statistical Analysis

##### Semiquantitative Analysis

Integral intensities of selected IR bands in the second-derivative spectra were calculated using the Opus software, based on 100 raw spectra per experimental group randomly extracted from the tumor area. Then, their box charts were constructed. A one-way analysis of variance was performed using an ANOVA model, and significance (*p*-values) was determined using Tukey’s test (OriginLab).

##### Histograms

Pixel spectra extracted from hyperspectral images were used to characterize the spatial distribution of spectral parameters within each tissue section; however, the biological unit of replication corresponds to the individual tissue sample, as spectra within the same sample are spatially correlated. Statistical analysis was performed on wavenumber distributions corresponding to selected spectral regions investigated here. Given the large number of spatially correlated pixel spectra in hyperspectral data sets, statistical significance was assessed alongside the magnitude and consistency of shifts in spectral parameter distributions. Each frequency map consisted of several thousand pixel-level spectra from tissue regions representing four tumor phenotypes: primary cancer in the mammary gland (PC), small cluster of cancer cells (SC), EMT perivascular tumor (EMT), and solid tumor in the parenchyma (ST). The resulting pixel-level distributions visualized as histograms were used to calculate the mean wavenumber (ν̅), standard deviation (SD), and standard error of the mean (SEM). To assess differences between groups, an ANOVA method was applied, followed by Tukey’s HSD *post hoc* test for multiple pairwise comparisons. The significance level was set to at least *p* < 0.05. Statistical parameters, including the means, standard deviations, F statistics, degrees of freedom, and Tukey’s pairwise comparisons, are summarized in Table S1 in the SI.

## Results and Discussion

### 4T1 Cells before and after Inoculation Into the Mammary Gland

First, SD IR imaging and 2-class UHCA analysis were employed to obtain a spectral characterization of the 4T1 cancer cells (Figure S2 in SI). In total, 15 IR images, each from an area of 700 μm x 700 μm, captured hundreds of the cells that formed clusters (Figure S2A in SI). One UHCA class and its FTIR spectrum represent the collective biochemical information on the whole cells (Figure S2C and Table S2 in SI). Next, we compared this FTIR spectrum with a spectrum of the primary tumor in the mammary gland to investigate the chemical alterations of the 4T1 cells due to their settling and intercellular interactions within the gland matrix (Figure 2C–E).

**Figure 2:**
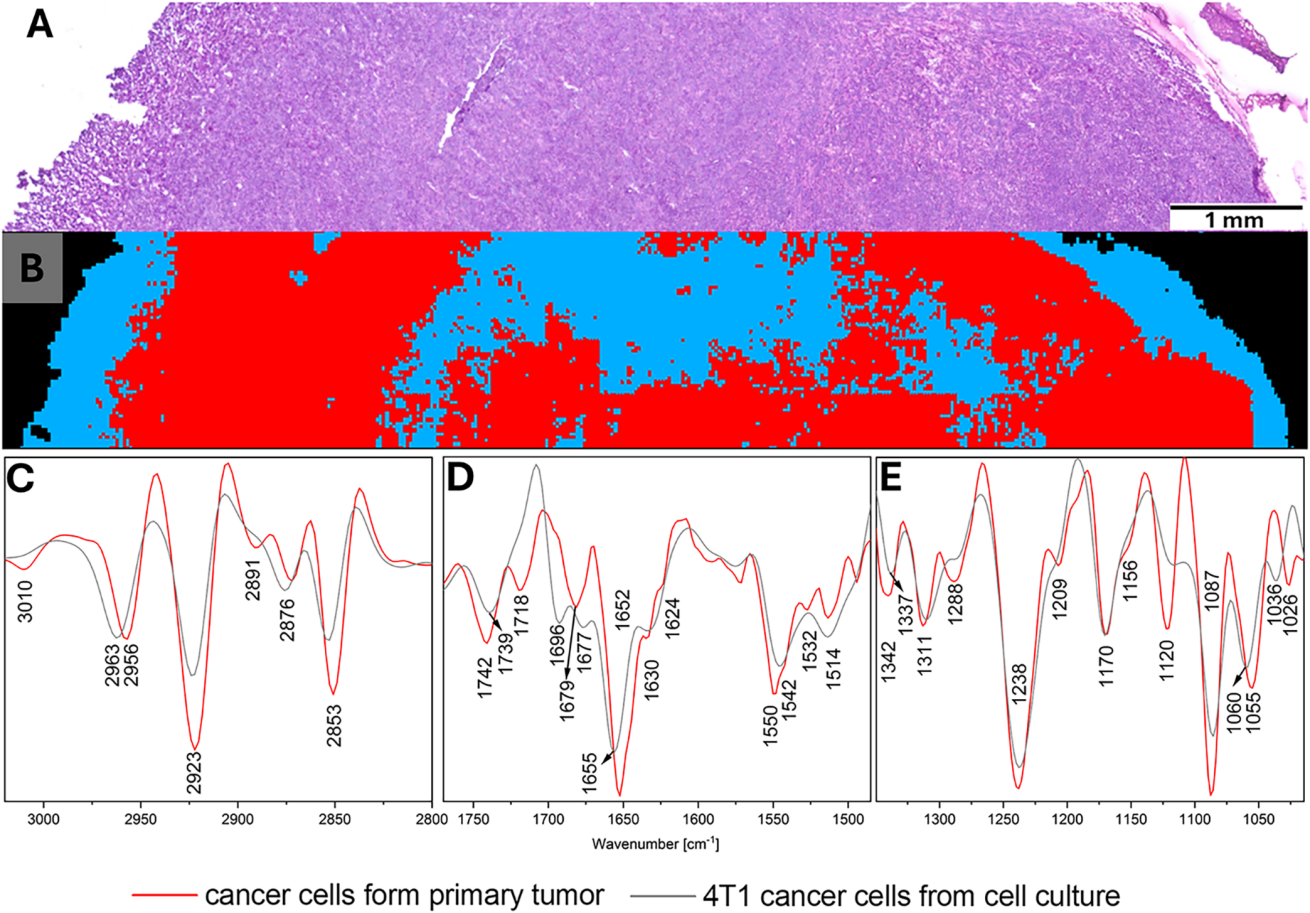
(A) H&E staining of OCT-frozen cross-section of the primary tumor in the mammary gland and the corresponding false-color UHCA map (B) showing the distribution of the cancer cells (red, the tumor of high density). (C–E) Secondary derivative FTIR spectra of cancer cells in the tumor (red trace) and the 4T1 cells from *in vitro* culture (gray trace, from Figure S1C in SI). The 1087 and 1055 cm^–1^ bands may include OCT vibrations.^17^

For this purpose, we prepared a tumor cross-section using the OCT cryo-sectioning method, as it has a negligible effect on the biochemical composition of the tissues. H&E staining reveals tight connections between the cancer cells throughout the entire volume of the primary tumor, which has a diameter of approximately 8 mm. At the same time, UHCA analysis of the IR image categorizes it into two classes based on tumor density (Figure 2A,B). The spectral differences between the two classes are negligible (data not shown). The intercellular interactions in the primary tumor substantially modify lipid metabolism. An increase in the lipid content in the tumor (2853 and 2923 cm^–1^) is accompanied by the synthesis of unsaturated fatty acids (3010 and 1718 cm^–1^); see Figure 2C,D, Table S2 in SI. In turn, the amide I region (1600–1700 cm^–1^) indicates changes in secondary structures of proteins in the tumor, and most of them form α-helices (1652 cm^–1^) and intramolecular aggregates (1679 cm^–1^) compared to the complex protein conformations in the single cells as the several Amide I bands are observed in the 1600–1700 cm^–1^ region (Figure 2D).

### Migration of Cancer Cells from the Mammary Gland to the Lung

The FFPE fixation method is preferable in histopathology because it preserves tissue morphology well. For this reason, we used this method for subsequent hyperspectral imaging of the tumor in the mammary gland and metastatic foci in the lungs. Although FFPE causes protein cross-linking, the deparaffinization of the tissue cross sections removes most lipids, which is necessary before IR imaging. Despite this, the comparison of FTIR imaging results does not indicate significant differences in the clustering of IR images and extracted FTIR spectra, due to the low lipid content in the lung, as we previously observed in the vibrational spectra of the tissue homogenate (Figure S3 in SI).^18^ The spectral analysis must be limited to the fingerprint region (900–1800 cm^–1^) because of the paraffin residue in the high-wavenumber region (above 2800 cm^–1^) (Figure S1 in SI). Despite this, the key cells and compartments of the tissue are well segregated by their unique chemical signatures, as we have shown in previous works.^12,17,19,20^ Here, the FTIR spectra of OCT and FFPE tissue cross sections for the primary tumor mainly differ by the intensity of the 1742 and 1200–1300 cm^–1^ bands assigned to triacylglycerols and collagens, respectively, and the presence of the OCT residue (Figure S3 in SI).

The formation of massive solid tumors in the mammary gland is readily recognized by histopathologists (Figures 3A and S4A in SI). The primary tumor exhibits the typical stromal progression with an inner necrotic core surrounded by homogeneous concentric proliferative regions. Due to metastasis to the lung, the single cancer cells first infiltrate the lung parenchyma. They are barely noticeable in the H&E microphotograph because their size within the tissue matrix does not exceed 10 μm (Figures 3B and S4B in SI). While they clump together to form small clusters, a high nucleus-to-cytoplasm ratio, specific to the cancer cells, is detectable among other cells (Figures 3C and S4C in SI).

**Figure 3:**
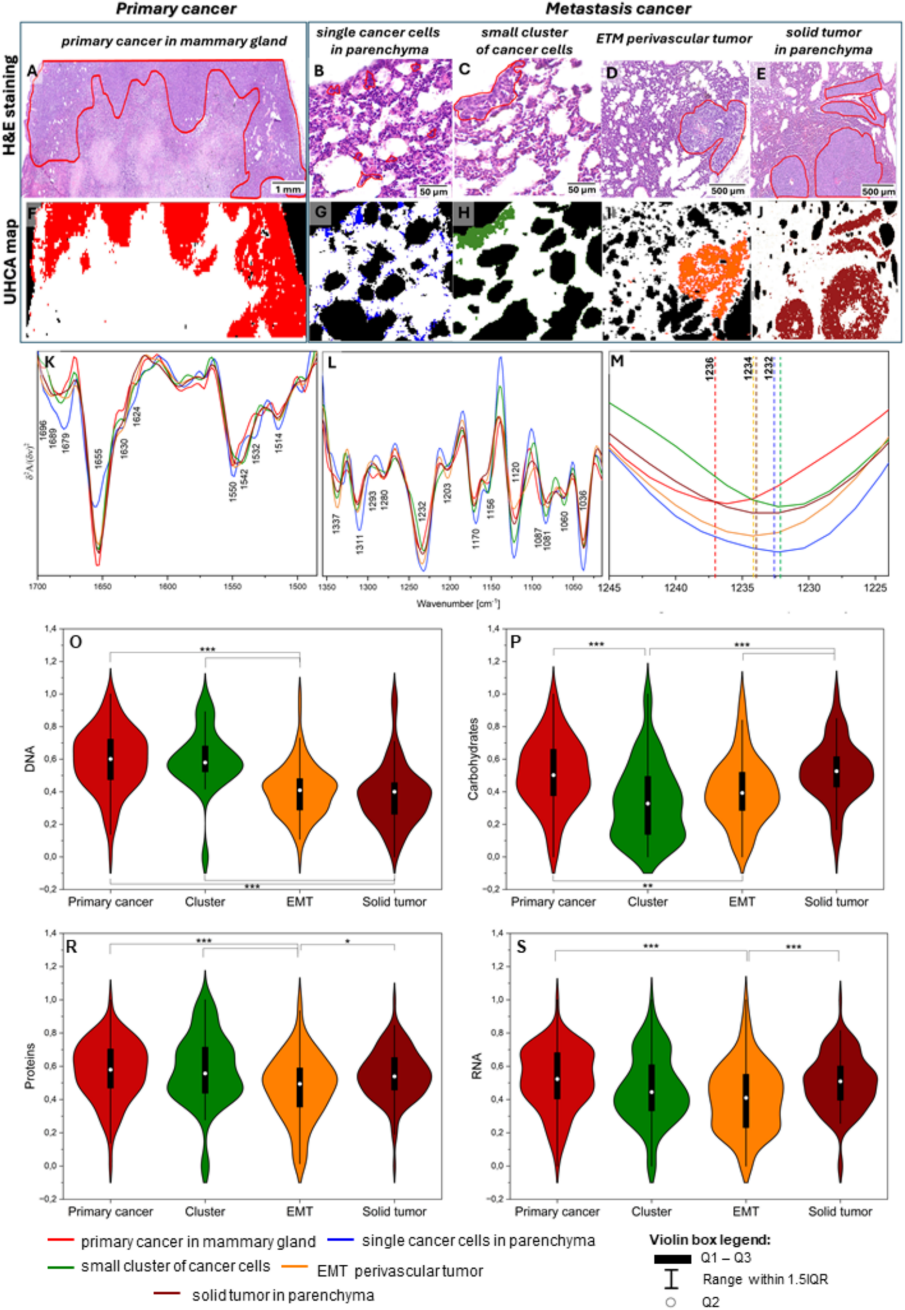
Localization of cancer cells in the mammary gland and lungs in the FFPE tissue cross sections in H&E microphotographs (A–E, enlarged microphotographs are given in Figure S4 in SI) with red outlined cancer cells. False-color UHCA maps with color-coded cancer class (F–J) corresponding to displayed spectra (K–M). Mean FTIR spectra of all UHCA classes are shown in Figure S5 in SI. Estimated FTIR indicators of biochemical changes in cancer cells: (O) DNA (band **–** 1232 cm^–1^), (P) carbohydrates (band **–** 1036 cm^–1^), (R) proteins (band **–** 1655 cm^–1^), and (S) RNA (band **–** 1120 cm^–1^) calculated from 100 spectra randomly chosen from the tumor areas. One-way ANOVA was used to determine statistical significance at *p*-value thresholds of *p < 0.05, **p < 0.01, and ***p < 0.001.

At an advanced stage of metastasis, cancer cells can undergo EMT to initiate metastasis to other organs. In this phase, the cancer cells become elongated, increasing their migratory capacity, invasiveness, and resistance to apoptosis (Figures 3D and S4D in SI).^21^ The solid secondary tumor is morphologically similar to that in the mammary gland (Figures 3E and S4E in SI). In each case studied here, label-free hyperspectral IR imaging combined with clustering reproduced the localization of the cancer cells regardless of their size and surrounding cells (Figure 3F–J). Their margins in H&E and UHCA differ slightly in the stroma, which constitutes the border between the tissue and the cancer cells, and these differences were identified only in H&E. This means that UHCA perfectly discriminates the cancer cells, excluding the surrounding microenvironment composed of extracellular matrix, fibroblasts, and infiltrating cancer cells. First, we examined the FTIR spectra of the primary and metastatic cancer cells obtained by averaging numerous pixel spectra assigned by UHCA clustering in Figure 3F–J. Each phase of metastasis differs spectrally from the primary tumor, and the most pronounced changes are observed in the lung infiltration by cancer cells (Figure 3G,K–M). Detailed interpretation of the spectral differences between lesions in this animal model was published in our previous articles.^11,22,36^ Moreover, based on the integral intensities of selected bands, we estimated FTIR indicators of biochemical changes in metastatic cancer (Figure 3O–S), which are discussed below.

### Biochemical Heterogeneity within the Primary Tumor and Metastatic Foci–Frequency Maps

In previous works on FTIR-based identification of cancer type and metastasis, band occurrences and shifts assigned to biomolecules were observed during the progression of the cancer disease.^13,22–25^ An example of these spectral alterations is also evident in the position of the asymmetric stretching vibration of the phosphate group, indicating intergroup variability among cancer lesions at various growth phases (Figure 3M). However, one must be aware that generating the mean spectrum for the cluster in CA can average different frequency positions, thereby losing information about their variation. For this purpose, we calculated 3D frequency maps to visualize the chemical heterogeneity within cancer cell clusters, accounting for genetic (1246–1222 cm^–1^), proteomic (1692–1672, 1170–1140, and 1074–1048 cm^–1^), and metabolic (1074–1048 cm^–1^) diversity (Figures 4–6). They exhibit the localization of selected band positions in pixels of the tumor IR image, indicating molecular and structural changes in the investigated classes of biomolecules between tumors at their origin and during growth. Histograms show density estimates, demonstrating the spectral regions of frequency positions and the distribution of frequency bins, and were constructed at 1 cm^–1^ intervals.

**Figure 4:**
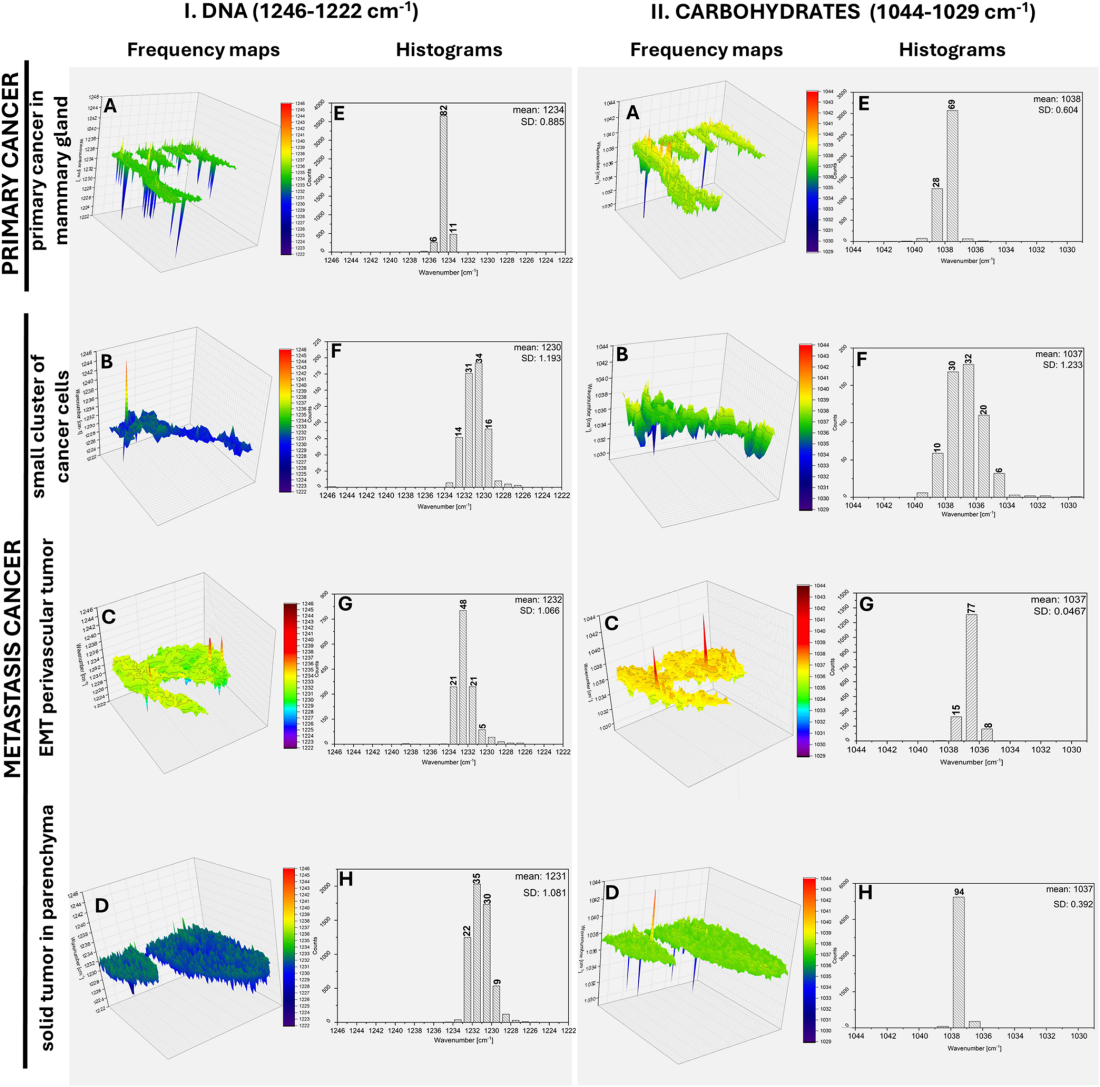
3D frequency maps (A–D) and their histograms (E–H) for DNA (panel I, the asymmetric stretching mode of the PO_2_^–^ group, 1246–1222 cm^–1^ region) and carbohydrates (panel II, the stretching modes of the C–O and C–C moieties in the ring, 1044–1029 cm^–1^) calculated for the FFPE cross sections of primary tumor and metastatic foci in the lung. Numbers in the histograms denote a particular frequency’s percentage contribution ≥5%.

First, we compare frequency maps calculated with interpolation densities of 8 and 16, as well as absorbance and second-derivative IR spectra of the solid tumor in the lung parenchyma in the 1222–1246 cm^–1^ region (Figures 3J and S6 in SI). The maps and their histograms differ, highlighting this approach’s sensitivity to the numerical factors used (Figure S6A,B1–4 in SI). Reducing the interpolation density (from 16 to 8) leads to artificial broadening due to coarser sampling and less precise band localization. Higher interpolation density increases the number of sampling points along the spectral axis, reducing discretization effects and improving the precision of band-position determination at the pixel level. In turn, the use of the second-derivative spectra enhances the resolution of overlapping and broad bands and reduces the baseline variations and artifact-related effects. In addition, the most abundant frequencies agree well with the band positions only in the case of the second-derivative spectra (Figure S6C1–4 in SI). Overall, the selected parameters (interpolation of 16 and second-derivative spectra) provide improved precision in band localization, better resolution of overlapping spectral features, and reduced numerical artifacts, making it the most reliable and consistent strategy for frequency map analysis within the scope of this study. One must be aware that the frequency maps require absorbance IR spectra with a high signal-to-noise ratio and free of vapor absorption, as the second derivative band shape is inversely proportional to the square of the original band half-width, and an enhancement of the contribution of sharp lines due to noise and vapor is highly probable.

The next evidence, confirming the specificity of frequency distribution to cancer cells, is provided by the frequency maps and histograms generated for three different ROIs (Region of Interest) around the solid tumor in the lung parenchyma (Figures 3J and S7 in SI). We intentionally selected the regions in the IR image, which include the tumor, stroma, and surrounding tissue (ROI 1, Panel 1A,B in Figure S7) and the tumor center (ROI 3, Panel 3A,B in Figure S7), comparing both to the tumor without stroma discriminated by IR image clustering (ROI 2, Panel 2A,B in Figure S7). The ROI 1 frequency map and its histogram show a very broad frequency distribution (mean: 1230 ± 5.4 cm^–1^), reflecting substantial heterogeneity in the 1222–1246 cm^–1^ region due to the presence of surrounding tissue, in contrast to ROIs 2 and 3 (1231 ± 1.1 and 1233 ± 0.9 cm^–1^, respectively). The latter are very similar, given that ROI 3 is more detailed than ROI 2. This confirms a reproducible map generation for the targeted tissue structure–cancer cells.

We excluded single cancer cells in the lungs from this analysis (Figure 3 B,G), since the frequency maps showed only slight variation (data not shown), and the most frequent band positions corresponded to those in the mean FTIR spectrum (Figure S5B in SI). Also, given the large number of pixel spectra (approximately 12,000–13,000 per tumor cluster), we determined the F statistics for each tumor class; the differences are highly significant (*p* < 0.0001), confirming that this analysis discriminates between tumor phenotypes (Table S1 in SI).

The wavenumber centered at ca. 1230 cm^–1^ is assigned to the asymmetric stretching mode of the phosphate groups. It is a well-established DNA marker, and its shift indicates a transition in DNA conformation between the A (1240 cm^–1^) and B (1220 cm^–1^) forms, likely due to chromatin folding induced by the microenvironment, different proliferation rates at various tumor sites, necrosis in the center of large cancer lesions, or somatic mutations in the cancer genome.^26–28^ The UHCA spectra already reveal DNA conformational changes in the cancer progression, since the position in the spectrum of the 4T1 cells before inoculation into mice is 1238 cm^–1^, shifts to 1236 cm^–1^ in the primary tumor and to 1232 cm^–1^ in the lung lesions (Figure 3M).

The frequency maps in the 1246–1222 cm^–1^ region show inter- and intragroup variability (Figure 4I). In the mammary gland tumor (465 × 10^3^ μm^2^), this variation is slight, i.e., 82% of pixels exhibit the 1234 cm^–1^ position (DNA A). The remaining ones (16.5%) differ by 1 cm^–1^ only (Figure 4I A,E). This implicates a lack of pronounced molecular diversity of DNA in the primary tumor. Significantly, all frequency maps for the metastatic foci show a downshift of this band up to 1226 cm^–1^, indicating a transformation of the DNA structure toward the B form. The pattern of the distribution of this variable becomes wider and right-skewed with the growth of the secondary tumor (Figure 4I F–H). The most frequent DNA positions (>5%) are centered in the 1229–1233 cm^–1^ range, as seen in the FTIR spectrum of the single cancer cells detected in the lungs (Figures 4 I–F and S4B in SI). Only the 1233 cm^–1^ frequency appears in the primary tumor and metastasis foci, which might be correlated with the primary origin of these cancer cells since an effect of the microenvironment is rather excluded. Next, tumor progression with a large size and high metastatic potential to distant organs induces intratumor heterogeneity in the DNA position, without locating specific frequencies in the tumor center, which can be associated with the necrotic core (Figure 4I G,H). The frequency maps have no unique distribution pattern (Figure 4I A–D). All frequencies are unevenly distributed across pixels, indicating that cells with a particular DNA frequency are neither clustered nor located at specific sites within tumors. We also examined the region of the symmetric stretching of the phosphate group in terms of its suitability for DNA evaluation. The frequency maps and histograms in the 1074–1088 cm^–1^ region showed 6–10 cm^–1^ variability, difficult for interpretation, likely due to the co-occurrence of C–O modes of carbohydrates and matrix proteins (data not shown).

We also estimated DNA levels as an indicator of nuclear volume in the metastatic foci. The values remain comparable between primary cancer and early micrometastasis; however, DNA content declines in EMT (Figure 3O). This demonstrates that the DNA phenotype of the primary tumor is preserved during early metastasis cluster formation, whereas it undergoes modifications during the EMT process, which are subsequently maintained in the solid tumor. Furthermore, min-max values also confirmed broader variability in nuclear dimensions in the primary cancer and the lung cancer cluster (ca. 0.4–0.8) compared to the EMT and solid tumors (ca. 0.1–0.5).

The stretching and deformation vibrations of the C–OH moiety in monocarbohydrates are observed in the 900–1100 cm^–1^ region and have been correlated with the glycolytic pathway in cancer malignancy and metastasis, as demonstrated in an IR study on plasma from this animal model of breast carcinoma.^13,29^ The frequency maps for the carbohydrate bands in the 1044–1029 cm^–1^ range display a similar distribution, with the predominant frequency at 1037 cm^–1^ band for primary tumor in the mammary gland (69%) and the solid one in the lung (94%) implicating that the cancer cells in the large tumors exhibit a similar metabolic adaptation (Figure 4II E and H). Approximately 30% of high-frequency pixels in mammary cancer are accumulated in the necrotic core (Figures 3A and 4II A). During lung colonization, the clustered cancer cells on the pleura exhibit the most pronounced carbohydrate diversity, as evidenced by band positions ranging from 1034 to 1039 cm^–1^ (Figure 4II B and F). This is expected since cancer cells must reprogram their energy metabolism and proliferation to adapt to the new environment and can be exposed locally to varying nutrient levels. Only 30% of the pixels display positions like those in the primary tumor. The others are associated with dysregulation of fructose metabolism and an increased glycolytic rate, including several intermediates such as hexose-P, fructose-1,6-bisphosphate, or pentose-5-phosphate, specific to colonizing cancer cells.^29,30^ Less heterogeneity of the carbohydrate bands is observed in EMT, i.e., in the 1037–1035 cm^–1^ range (Figure 4II C and G). The dominant frequency at 1036 cm^–1^ (ca. 77% of pixels) is the same as in the other large tumors (Figure 4II E and H). In contrast, the high-frequency pixels (ca. 15%) are located around the blood vessel – a potential site of further metastasis of the EMT cancer cells, where they are less compacted (Figure S4 D in SI). In the case of EMT, O-glycosylation of Ser and Thr residues in proteins is upregulated, causing the secretion of proteins and cell-surface mucins. One of their IR characteristic bands is centered at around 1040 cm^–1^ in reference spectra and can be attributed to these high-frequency pixels.^31,32^ The content of carbohydrates also varies between the metastatic phases (Figure 3P). As shown in the frequency maps, clusters formed during early metastasis exhibited the greatest carbohydrate diversity, with a broad range of their content (ca. 0.1–1.0) and heterogeneous spatial distribution, clearly distinguishing this stage from the others. In contrast, subsequent metastatic transformations exhibited a gradual increase in carbohydrate levels, with EMT (approximately 0.2–0.6) and solid tumors (approximately 0.4–0.8). Interestingly, the carbohydrate distribution in primary tumors appeared as a composite pattern, encompassing features characteristic of both EMT and solid tumors.

The development of invasive tumors and their metastatic dissemination alter the local extracellular matrix (ECM), leading to the cross-linking and stiffening of collagen-rich fibrils, fibronectin, proteoglycans, and other components. They facilitate the migration, intravasation, and extravasation of cancer cells.^32^ Since we cannot distinguish the cells from the ECM due to the low resolution of the IR microscope, the proposed descriptors of the protein pool illustrate their entire modifications in the area occupied by the cancer cells. We calculated the intensity of the amide I region to estimate the total content of proteins as an indicator of cell proliferation (Figure 3R). For pleural cluster, the protein content was distributed into two distinct ranges (0.3–0.5 and 0.6–0.8), and a bimodal distribution was also observed for carbohydrates (Figure 3P). Such division confirms an excessive metabolic demand during colonization of the new organ. Interestingly, the EMT shows the lowest protein level, which differs statistically from other cancer formations. The determined levels of protein synthesis agree well with the IR signal of RNA that supports protein production through transcription and processing (Figure 3S). Furthermore, an analysis of the amide I region reveals only variation in the absorbance maxima assigned to turns (approximately 1688 cm^–1^) and intramolecular aggregates (1680 cm^–1^), as shown in Figure 3K. The 1680 cm^–1^ band also serves as an indicator of collagen fibril formation.^33,34^ α-Helice and β-sheet bands at ca. 1650 and 1630 cm^–1^, respectively, are not shifted in the tumors (Figure 3K). The distribution patterns of the 1688 and 1680 cm^–1^ bands indicate the remodeling of protein structures (Figure 5).

**Figure 5:**
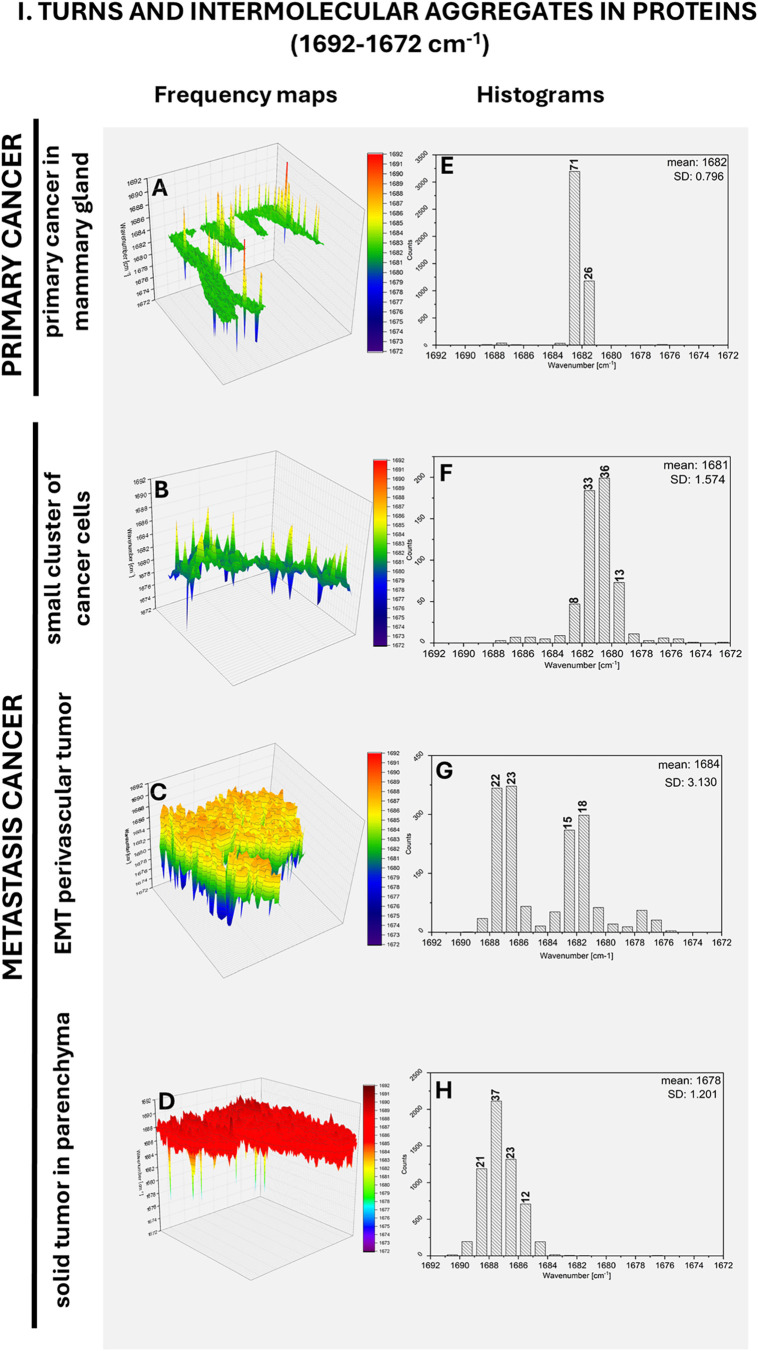
3D frequency maps (A–D) and their histograms (E–H) for the amide I band in the 1690–1672 cm^–1^ region calculated for the FFPE cross sections of primary tumor and metastatic foci in the lung. Numbers in the histograms denote a particular frequency’s percentage contribution ≥5%.

Most primary tumor proteins aggregate (71% pixels at 1682 cm^–1^), and this process is maintained in the small cluster of cancer cells. However, the aggregated protein structures are more diverse, as shown by a broad region of the frequencies (1682–1679 cm^–1^) (Figure 5F). Surprisingly, the EMT tumor is characterized by two amide I maxima at ca. 1687 and 1682 cm^–1^, which are attributed equally to each image pixel (50:50%). Integral intensities also capture this differentiation, highlighting the ETM as the most distinct among cancer formations in terms of their proteomics (Figure 3R). Their distribution patterns resemble those of cancer cells (1683–1676 cm^–1^) and a solid tumor in the lung parenchyma (1690–1684 cm^–1^) (Figure 5F–H). The amide I marker of the protein aggregates disappears in the latter (Figure 5H).

In our previous work on an intravenous animal model of mammary gland carcinoma, we have established a correlation between the IR bands of hydroxyproline residues (Hyp) of collagens (ca. 1155 cm^–1^) and elastin (ca. 1060 cm^–1^) and the degradation of ECM in cancer-driven endothelial dysfunction in aged individuals.^14^ The Hyp residues play a crucial role in stabilizing the collagen triple helix and participate in H-bonding, facilitating the formation of fibrils and the polypeptide backbone stiffness, enabling the migration of the cancer cells in ECM.^35^ Unfortunately, details on structural changes in elastin have not yet been reported. Calculated 3D frequency maps in the corresponding spectral regions reveal two major frequency groups in each case (Figure 6).

The prominent frequencies at 1155 and 1060 cm^–1^ are accompanied by bands at ca. 1163 and 1072 cm^–1^ for Hyp and elastin, respectively, except for the IR spectra of the small cluster of the cancer cells (Figure 6E–H, panels I and II). We assumed that this splitting resulted from the structural deformations of these scaffold proteins, which are specific to each clumping of the cancer cells, because the reference IR positions in the spectra of neat collagen I/III/IV as well as elastin, are only observed at 1160 and 1060 cm^–1^, respectively.^36^ The Hyp frequencies vary little, but their pixel ratio (ca. 3:1) is similar for the metastatic tumors (the primary and EMT tumors) and different for the initial phase of the lung invasion and the large solid tumor, i.e., collagens are insignificantly remodeled in the former and completely degraded in the latter as expected (Figure 6E–H, panel I). The frequency maps show a uniform distribution of this process in contrast to the remodeling of the elastin fibrous.

**Figure 6:**
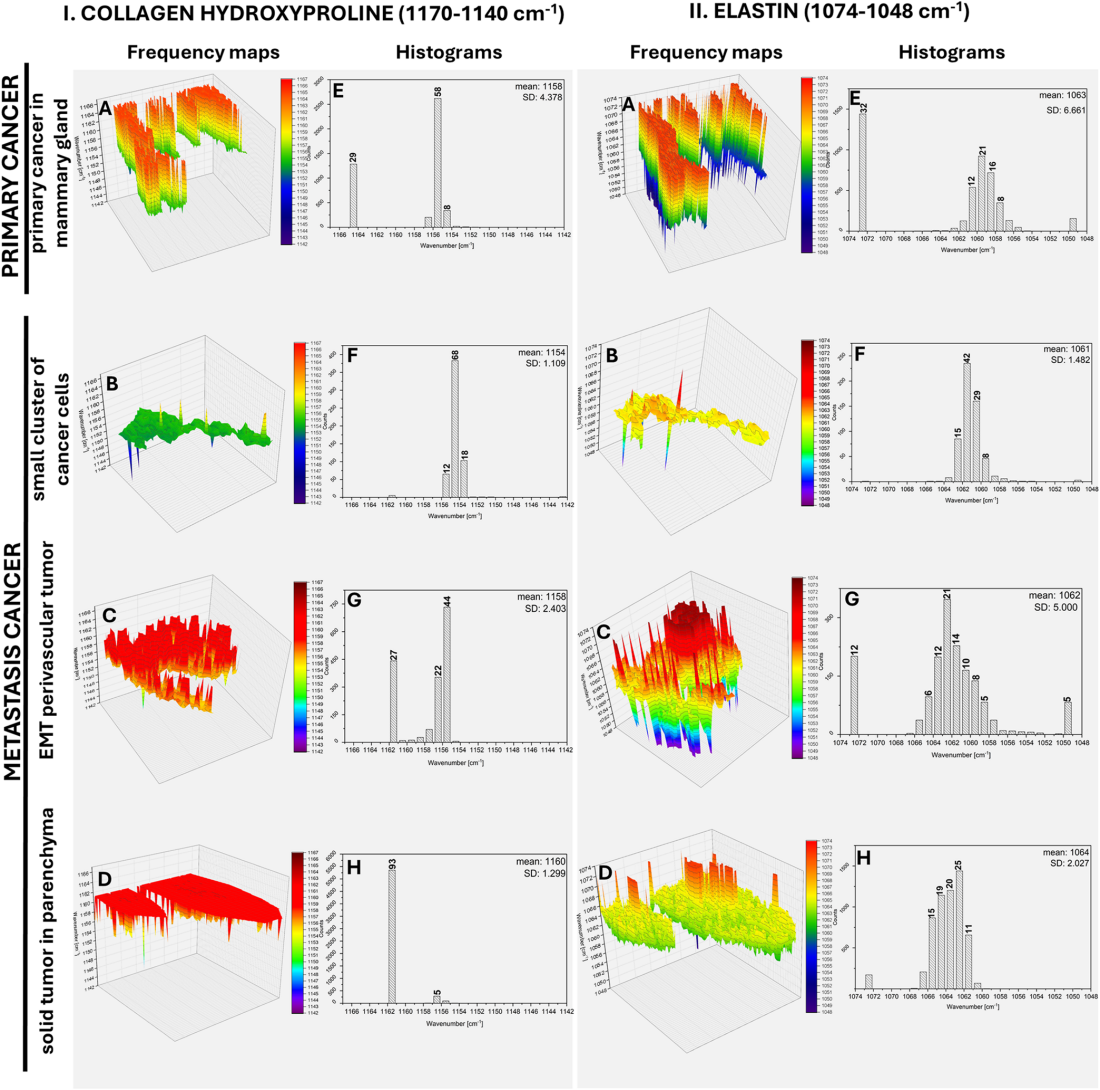
3D frequency maps (A–D) and their histograms (E–H) for the bands of collagen Hyp (1170–1140 cm^–1^, panel I) and elastin (1074–1048 cm^–1^, panel II) calculated for the FFPE cross sections of primary tumor and metastatic foci in the lung. Numbers in the histograms denote a particular frequency’s percentage contribution ≥5%.

Compared with the frequency maps presented in Figures 4 and 5, the additional data sets show a similar trend across both the DNA and protein spectral regions (Figures S8–S9 in SI). Smaller tumors exhibit more homogeneous spectral characteristics, reflected by higher mean band positions in the DNA range (∼1233–1234 cm^–1^) and narrower histogram distributions with well-defined maxima in the protein region (∼1681–1682 cm^–1^). In contrast, larger tumors display lower mean values in the DNA region (∼1231–1232 cm^–1^), and broader, more dispersed histograms in the protein range, with dominant band positions shifted toward lower wavenumbers (∼1678–1680 cm^–1^) and less distinct maxima. These observations indicate that increasing tumor size is associated with greater biochemical and structural heterogeneity, resulting in more complex and spatially variable spectral signatures. However, to draw a definitive conclusion about the statistical significance of such a correlation, an extensive study must be performed, including lesion localization and size in IR imaging, and reference bioassays that characterize genetic variability and metabolic activity of single cancer cells within the tumors.

## Conclusions

This work demonstrates the proof of concept that spectral histopathology, employing FTIR spectroscopy, can be extended to detailed investigations of molecular variability induced by cancer growth and metastasis. Hyperspectral FTIR imaging, combined with unsupervised machine learning approaches, detected single cancer cells, tumors, and their margins with sizes ranging from 10 to 10,000 μm^2^. As has been shown many times, this result can be further used to support clinical histology in identifying carcinomas and assessing their malignancy. Here, we demonstrate that frequency maps in specific spectral regions provide additional information about molecular diversity that depends on the tissue environment and tumor mass, and localize this diversity within various structures. The frequency histograms quantify the contributions of molecular species across the classes of nuclear DNA, cytoplasmic and ECM proteins, and carbohydrate-related metabolism. The chosen DNA spectral signal could be assumed to be an indicator of genetic events, as it is sensitive to DNA structure and its alterations due to pronounced mutations and methylation, which are expected in each cancer. However, further validation, preferably by genetic tests, is required to understand these changes. Here, the differences between lesions in the mammary gland and the lung become more pronounced with progressive colonization of the metastatic organ. The characteristic carbohydrate region highlights the pronounced metabolic reprogramming in malignant cells upon colonization of the distal organ. At that point, the cytoplasmic proteins are rearranged compared to the primary site of the carcinoma, and their frequency maps exhibit intriguing evolution of the protein pattern due to the tumor growth and mesenchymal transformation. The degradation of collagen and elastin in the ECM is also unique to the microenvironment and malignancy.

Undoubtedly, potential users of this method must be aware of the risk of false results arising from inappropriate data collection and preprocessing. The intratumor frequency shift, if present, can be hidden in the spectra of low spectral resolution, whereas a high signal-to-noise ratio is required to exclude the contribution of sharp lines due to noise and vapor. So, the effects of denoising and smoothing steps on the quality of the spectra should be carefully examined before the final interpretation of the frequency maps.

Further investigation is required to validate this method for animal cancer studies and clinical applications, thereby enhancing the identification of optimal spectral descriptors for assessing intra- and intertumor heterogeneity and enabling spatial and temporal monitoring. However, it is essential to consider the methodological and technological challenges associated with the use of genomics, proteomics, and metabolomics in cancer characterization, as well as the advantages of high-throughput single-frequency infrared spectroscopy using quantum cascade lasers. Despite these challenges, the potential for applying this spectral evaluation of tumors is substantial.

## Supplementary Material



## Data Availability

Raw measurement data are available here: 10.48733/IFJPAN/XZWCLD

## Notes

Statistical analysis, assignment of band positions, comparison of FTIR spectra, H&E images, and UHCA analysis (PDF)
