# Supplementary material for: Frequency Maps of FTIR Images for Phenotyping Molecular Heterogeneity of the Primary Tumor and Its Metastases
Source: Anal Chem. 2026 Jun 8;98(24):17591–603. doi: 10.1021/acs.analchem.5c05310 (PMC13308875; doi:10.1021/acs.analchem.5c05310)
Supplement: Supplementary file 1 [file ac5c05310_si_001.pdf]

Supporting Information

Frequency maps of FTIR images for phenotyping molecular heterogeneity of the primary tumor and its metastases

Karolina Chrabaszcz[1] *and Kamilla Malek*[2]*

[1] Institute of Nuclear Physics, Polish Academy of Sciences, Radzikowskiego 152, 31-342 Krakow, Poland

[2] Jagiellonian University in Krakow, Faculty of Chemistry, Gronostajowa 2, 30-38, Krakow, Poland

Corresponding Author: K. Malek; E-mail: kamilla.malek@uj.edu.pl

Table of Contents

Note S1. Pseudo-code for the algorithm.

Table S1. A summary of statistical analysis of wavenumber distributions.

Table S2. Band positions observed in infrared spectra with their assignment.

Figure S1. Comparison of FTIR spectra extracted from lung tissue sections prepared using the paraffin- and cryo-embedding methods.

Figure S2. IR results of 4T1 breast cancer cells culture**.**

Figure S3. The comparison of the second derivative FTIR spectra of the primary tumors from the FFPE and OCT-frozen tissue blocks.

Figure S4. Enlarged H&E images of the cancer cells in the mammary gland and the lungs.

Figure S5. UHCA analysis of the primary and secondary tumors.

Figure S6. Testing the generation of the frequency maps using different spectra and interpolation factors.

Figure S7. Testing the generation of the frequency maps for different ROIs of the metastatic foci.

Figures S8 and **S9.** Testing the generation of the frequency maps for different metastatic foci.

Note S1. Algorithm: Frequency map construction from hyperspectral FTIR data

Input:
- Hyperspectral FTIR dataset (pixel-wise spectra)
- Spectral resolution: 4 cm^{-1}
- Spectral sampling interval: ~1.9 cm^{-1}

Band-search windows:
- DNA: 1246–1222 cm^{-1}
- Carbohydrates: 1044–1029 cm^{-1}
- Protein turns / intermolecular aggregates: 1692–1672 cm^{-1}
- Collagen hydroxyproline: 1170–1140 cm^{-1}
- Elastin: 1074–1048 cm^{-1}

Preprocessing:
1. Exclude spectra with abnormal baselines or insufficient signal intensity by a quality test.
2. Compute second-derivative spectra using the Savitzky–Golay algorithm (13 smoothing points).

Bands localization (performed for each pixel spectrum):

3. For each predefined spectral window:
    a. Extract the spectral segment corresponding to the window.
    b. Apply cubic spline interpolation (interpolation factor = 16).
    c. Identify the minimum of the second-derivative band within the window.
    d. Record the wavenumber corresponding to the derivative minimum as the band position.

Frequency map construction:

4. Assign the obtained wavenumber value to the spatial coordinates of the pixel.

Output:
- Spatial frequency maps representing the distribution of band positions across the selected ROI.

Post-processing:

6. Compile band-position values from all pixels.
7. Visualize band-position distributions using histograms (bin width = 1 cm^{-1}).

Table S1. Summary of statistical analysis of wavenumber distributions corresponding to characteristic infrared bands (from Figs. 4 - 6).

	Group	N(pixels)	Mean wavenumber [cm^{-1}]	SD	SEM	Tukey pairwise comparison ($\Delta\nu$ [cm^{-1}], p-value)
**DNA**	PC	4482	1234.43	0.89	0.013	vs SC: +3.53, p<0.001<br>vs EMT: -2.25, p<0.001<br>vs ST: -3.24, p<0.001
	SC	561	1230.89	1.19	0.050	vs PC: -3.53, p<0.001<br>vs EMT: -1.28, p<0.001<br>vs ST: +0.18, p<0.001
	EMT	1656	1232.18	1.07	0.026	vs SC: +1.28, p<0.001<br>vs PC: +2.25, p<0.001<br>vs ST: -0.99, p<0.001
	ST	5760	1231.19	1.08	0.014	vs PC: +3.24, p<0.001<br>vs SC: -0.29, p<0.001<br>vs EMT: +0.99, p<0.001
	**Global test (one-way ANOVA)**					**F(3,12455) = 9048.83, p < 0.0001**
**CARBOHYDRATES**	PC	4482	1037.89	0.60	0.009	vs SC: -1.25, p<0.001<br>vs EMT: -1.24, p<0.001<br>vs ST: -0.45, p<0.001
	SC	561	1036.63	1.23	0.052	vs PC: +1.26, p<0.001<br>vs EMT: +0.02, p<0.001<br>vs ST: +0.79, p<0.001
	EMT	1656	1036.65	0.47	0.011	vs SC: -0.02, p<0.001<br>vs PC: +1.24, p<0.001<br>vs ST: +0.77, p<0.001
	ST	5760	1037.42	0.39	0.005	vs PC: +0.47, p<0.001<br>vs SC: -0.79, p<0.001<br>vs EMT: -0.77, p<0.001
	**Global test (one-way ANOVA)**					**F(3,12455) = 2566.84, p < 0.0001**
**TURNS AND INTRAMOLECULAR AGGREGATES**	PC	4482	1682.21	0.80	0.012	vs SC: -1.20, p<0.001<br>vs EMT: +2.00, p<0.001<br>vs ST: -5.00, p<0.001
	SC	561	1681.00	1.57	0.066	vs PC: +1.20, p<0.001<br>vs EMT: +3.20, p<0.001<br>vs ST: -6.21, p<0.001
	EMT	1656	1684.21	3.13	0.077	vs SC: -3.20, p<0.001<br>vs PC: -2.00, p<0.001<br>vs ST: +3.01, p<0.001
	ST	5760	1687.21	1.20	0.016	vs PC: +5.00, p<0.001<br>vs SC: +6.21, p<0.001<br>vs EMT: -3.01, p<0.001
	**Global test (one-way ANOVA)**					**F(3,12455) = 10 435.17, p < 0.0001**

	Group	N(pixels)	Mean wavenumber [cm^{-1}]	SD	SEM	Tukey pairwise comparison ($\Delta\nu$ [cm^{-1}], p-value)
**COLLAGEN and HYP**	PC	4482	1160.86	1.30	0.017	vs SC: +6.48, p<0.001<br>vs EMT: -3.35, p<0.001<br>vs ST: -0.07, p<0.001
	SC	561	1154.38	1.11	0.047	vs PC: -6.48, p<0.001<br>vs EMT: +3.13, p<0.001<br>vs ST: -6.56, p<0.001
	EMT	1656	1157.54	2.40	0.059	vs SC: -3.13, p<0.001<br>vs PC: +3.35, p<0.001<br>vs ST: +3.53, p<0.001
	ST	5760	1160.93	1.30	0.017	vs PC: +0.07, p<0.001<br>vs SC: +6.56, p<0.001<br>vs EMT: -3.42, p<0.001
	**Global test (one-way ANOVA)**					**F(3,12455) = 5644.56, p < 0.0001**
**ELASTIN**	PC	4482	1062.97	6.66	0.099	vs SC: +1.98, p<0.001<br>vs EMT: -0.74, p<0.001<br>vs ST: -0.90 p<0.001
	SC	561	1060.99	1.48	0.063	vs PC: -1.98, p<0.001<br>vs EMT: +1.24, p<0.001<br>vs ST: -2.88, p<0.001
	EMT	1656	1062,23	5.00	0.123	vs SC: -1.64, p<0.001<br>vs PC: +0.74, p<0.001<br>vs ST: -1.24, p<0.001
	ST	5760	1063.87	2.03	0.027	vs PC: +0.90, p<0.001<br>vs SC: +2.88, p<0.001<br>vs EMT: +1.64, p<0.001
	**Global test (one-way ANOVA)**					**F(3,12455) = 111.17, p < 0.0001**

Data are presented as mean wavenumber (cm^{-1}), ± SD (standard deviation), SEM (standard error of the mean), calculated from N pixel spectra within each tumor phenotype: PC (Primary cancer in mammary gland), SC (small cluster of cancer cells), EMT (EMT perivascular tumor), and ST (solid tumor in parenchyma). Tukey's HSD post hoc test was used to assess the statistical significance of differences in mean wavenumber between groups ($\Delta\nu$) at min. $p < 0.05$.

Table S2. Band positions observed in infrared spectra with their assignment to vibrational modes and biomolecules.

**Position [cm^{-1}]**	**Vibrational mode**	**Biomolecules**
3010	ν(=C-H)	Unsaturated fatty acids
2963	$\nu_{as}(CH_3)$	Mainly proteins, lipids
2923	$\nu_{as}(CH_2)$	Long acyl chain in fatty acids
2891	ν(CH)	Terminal CH_3 group in acyl chains
2876	$\nu_s(CH_3)$	Mainly proteins, lipids, nucleic acids
2853	$\nu_s(CH_2)$	Long acyl chain in fatty acids
1742	ν_{ester}(C=O)	Esters of fatty acids and phospholipids
1739	ν_{ester}(C=O)	Cholesterol esters
1718	ν(C=O)	Fatty acids
1696	Amide I: ν(C=O) and δ(N-H)	Anti-parallel β-sheets in proteins
1686-1690		Turns in proteins
1680-1684		Intramolecular aggregates in proteins
1655		α-Helices in proteins
1630		β-Sheets, triple-helices in collagens
1624		Intermolecular aggregates in proteins
1550	Amide II: δ(N-H) and ν(C-N)	Proteins (amide II)
1542		Proteins (amide II)
1532		Random scaffolds and cross-linking
1514	Ring ν(C=C)	Tyrosine residues, methylated DNA
1342	$\delta(CH_2)$	Long acyl chain in fatty acids, amino acid side chains
1340-1270	Amide III: ν(C-N), δ(N-H), and $\nu(CH_3\text{-}C)$	Proteins
1280, 1240, 1203	Amide III	Collages in ECM
1232-1240	$\nu_{as}(PO_2^-)$	Nucleic acids (mainly DNA), phospholipids, phosphorylated proteins
1170	ν_{as}(CO-O-C)	Cholesterol esters
1165 - 1156	ν(C-OH), δ(C-O-C)	Hydroxyproline residues in collagens
1120	ν(C-O)	RNA
1081	$\nu_s(PO_2^-)$	Nucleic acids, phospholipids, phosphorylated proteins
1070 - 1050	ν_{as}(CO-O-C)	Elastin
1036-1026	ν(C–O), ν(C–C)	Carbohydrates

ν – stretching mode, as – asymmetric, s – symmetric; δ – in-plane deformations; ECM – extracellular matrix.

The reader is referred to the reference list, in which the interpretation of FTIR spectra of cancer-related tissues is discussed in detail.:

Li, R.; Allen, H. C. Developing transferable and universal IR biomarkers for intraoperative colorectal cancer diagnosis via FTIR spectroscopy. *Sci. Rep.* **2025**, *15*, 22944. https://doi.org/10.1038/s41598-025-05068-z.

Galant, N.; Nicoś, M.; Khalavka, M.; et al. Application of Fourier transform infrared (FTIR) spectroscopy in liquid biopsy to predict the response to the first-line immunotherapy in non-small-cell lung cancer (NSCLC) patients. Biochem. Biophys. Res. Comm. **2025**, *771*, 152007. https://doi.org/10.1016/j.bbrc.2025.152007.

Chrabaszcz, K.; Kaminska, K.; Song S.L.; et al. Fourier Transform Infrared polarization contrast imaging recognizes proteins degradation in lungs upon metastasis from breast cancer, *Cancers*, **2021**, *13*, 162. https://doi.org/10.3390/cancers13020162.

Kujdowicz, M.; Perez-Guaita, D.; Chlosta, P.; et al. Evaluation of Grade and Invasiveness of Bladder Urothelial Carcinoma Using Infrared Imaging and Machine Learning. *Analyst* **2023**, *148* (2), 278–285. https://doi.org/10.1039/D2AN01583H.

Kujdowicz, M.; Placha, W.; Mech, B.; et al. In vitro spectroscopic-based profiling of urothelial carcinoma: A Fourier transform Infrared and Raman imaging study, *Cancers*, **2021**, *13*, 123. https://doi.org/10.3390/cancers13010123.

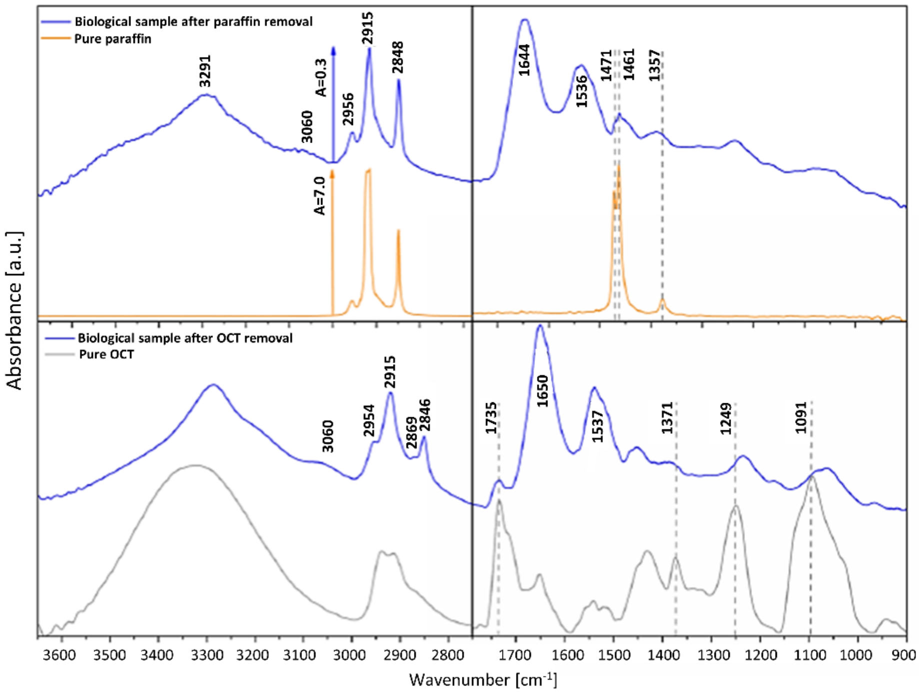

Figure S1. Comparison of FTIR spectra extracted from lung tissue sections prepared using the paraffin- (blue spectrum, upper panel) and cryo-embedding methods (blue spectrum, lower panel), together with the spectra of pure paraffin (yellow spectrum, upper panel) and OCT medium (gray spectrum, lower panel).

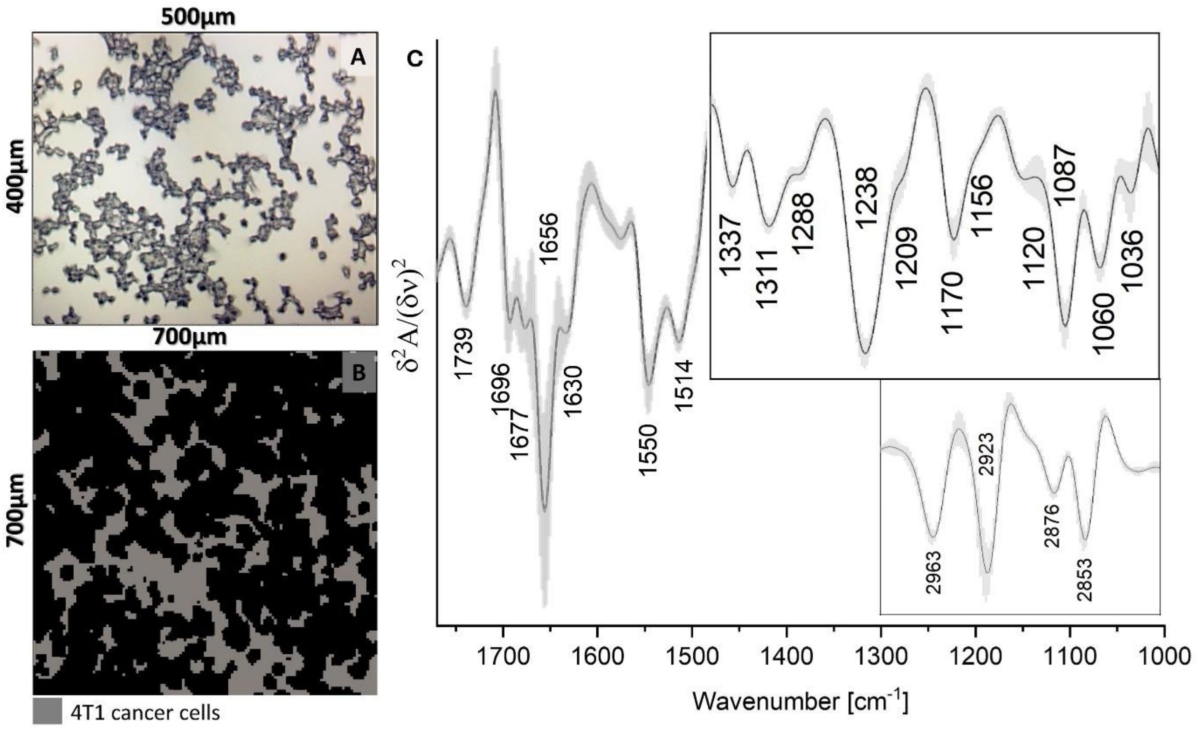

Figure S2. White-light image of 4T1 breast cancer cells culture (**A**, magnification 15x); a two-class UHCA map of IR image (**B**, gray – 4T1 cells, black – empty pixels) with the corresponding mean second derivative FTIR spectrum (**C**). Gray shading denotes standard deviation (±SD) calculated from 15 IR images (each from an area of 700 µm × 700 µm).

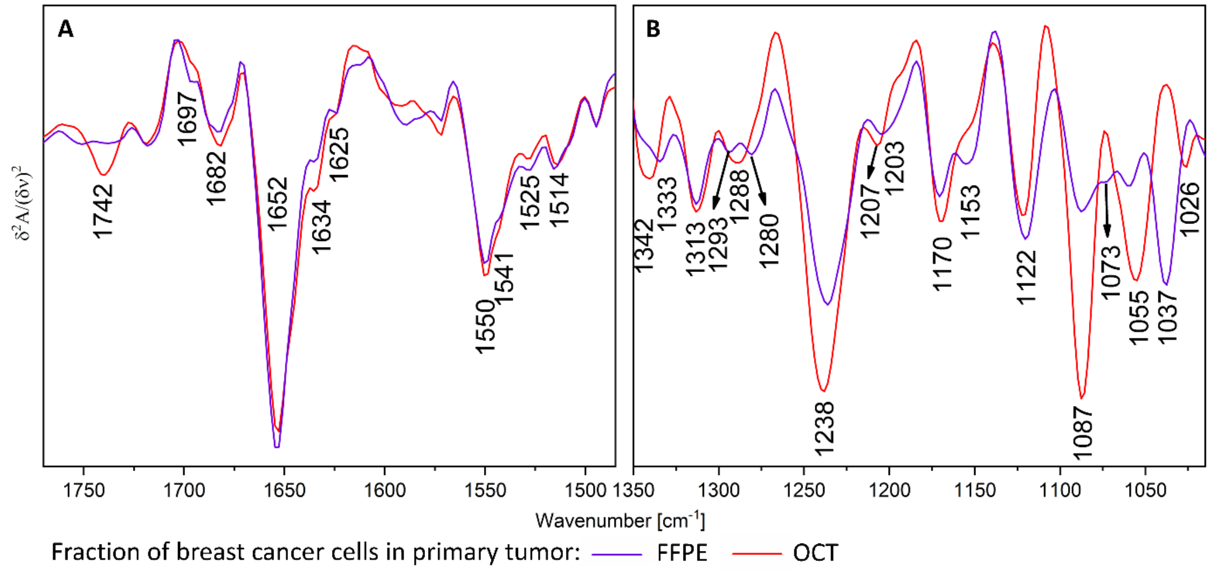

Figure S3. The comparison of the second derivative FTIR spectra of the primary tumors from the FFPE (blue trace) and OCT-frozen (red trace) tissue blocks. The 1087 and 1055 cm^{-1} bands may include OCT vibrations.

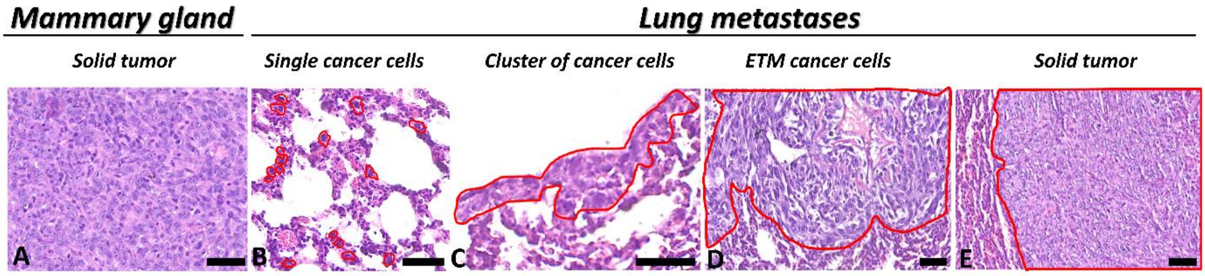

Figure S4. Enlarged H&E images of the cancer cells in the mammary gland (**A**) and the lungs (**B-E**, borders of the cancer cells marked in red). EMT – epithelial-mesenchymal transition. Scale bar is 50 μm.

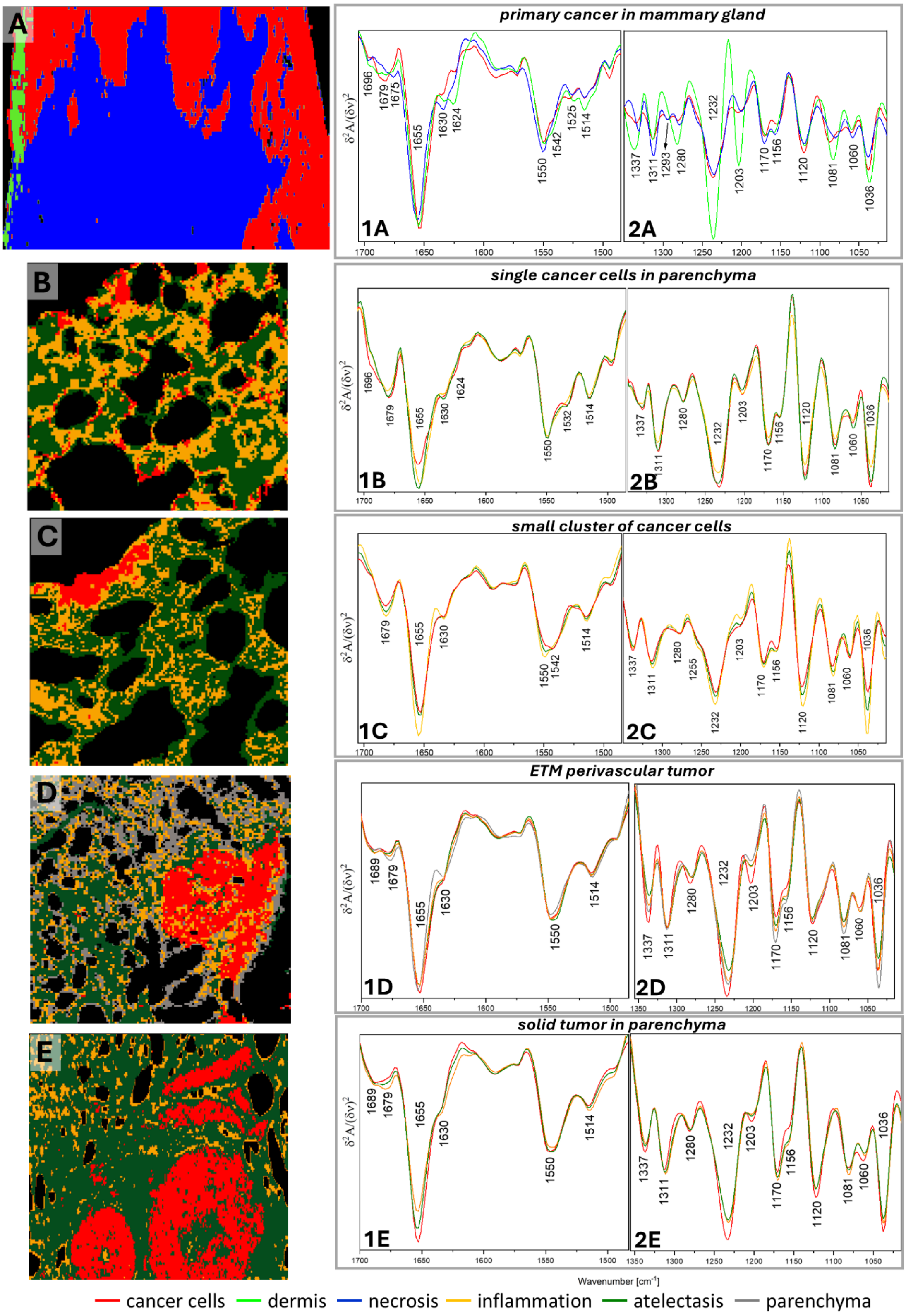

Figure S5. Mean second derivative FTIR spectra of classes from UHCA maps shown in **Fig. 3**. The colors of the spectra correspond to the colors of the UHCA classes. Color code: red – cancer cells, light green – dermis, blue – necrosis, dark green – atelectasis, yellow – inflammation, and gray – parenchyma.

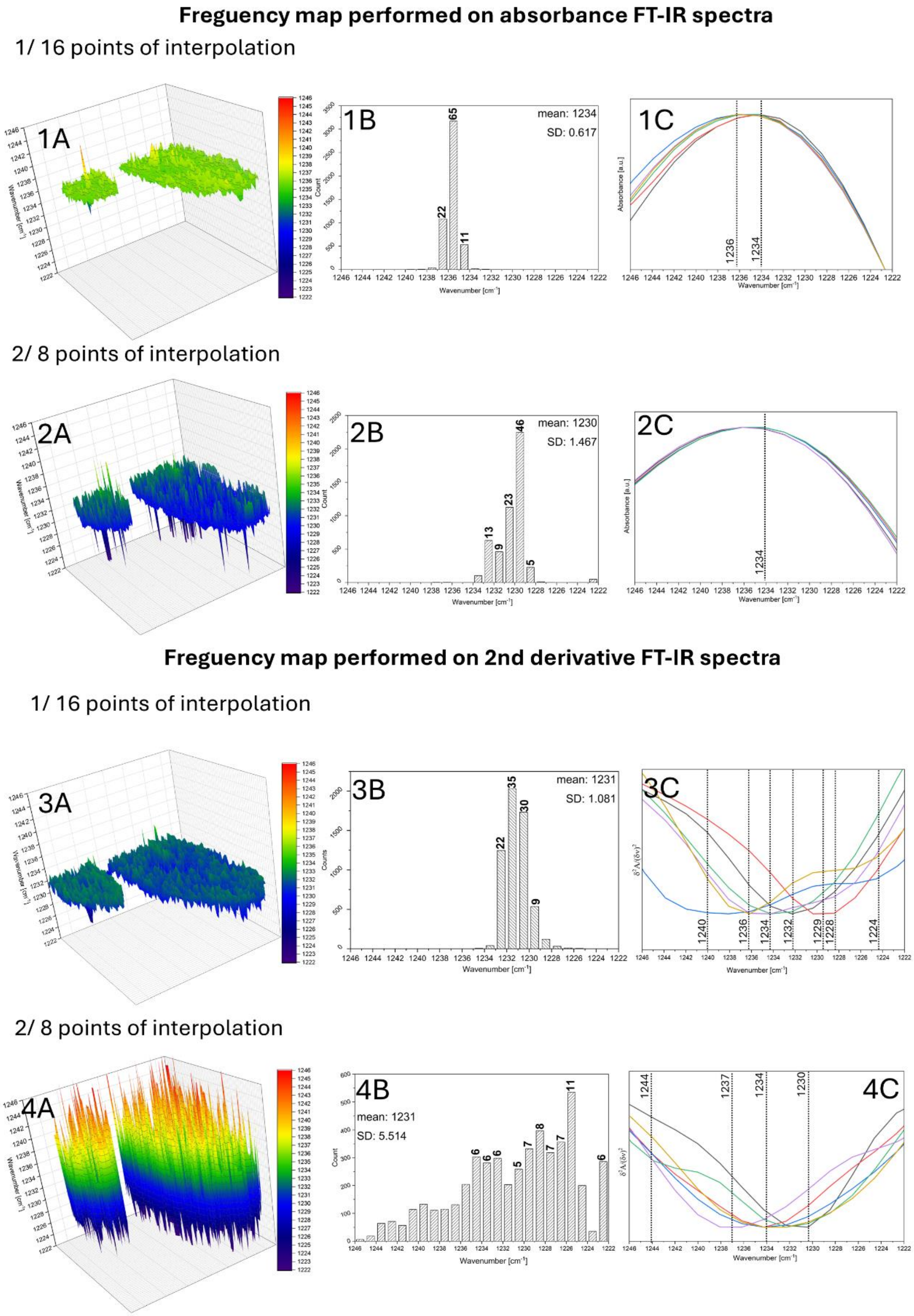

Figure S6. The comparison of the frequency maps (1A - 4A) and their histograms (1B – 4B) calculated with interpolation factors of 8 and 16 using absorbance and second-derivative FT-IR spectra for the class of the solid metastatic foci in the lung parenchyma shown in **Fig. 3 E and J**. (1C – 4C) The raw spectra were extracted from pixels corresponding to the most abundant frequencies, representing the most frequent spectral features within the analysed ROIs.

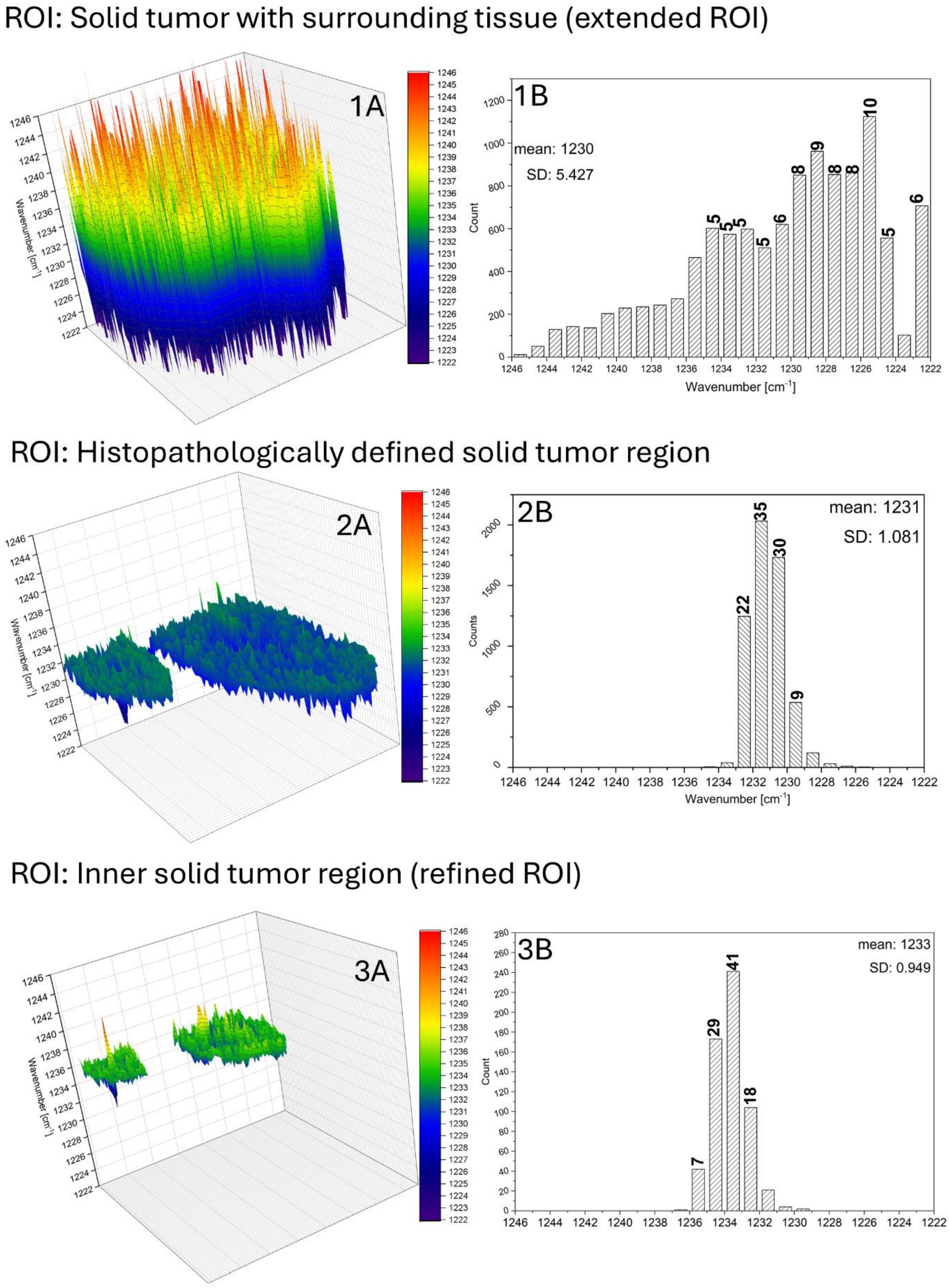

Figure S7. The comparison of the frequency maps (1A - 3A) and their histograms (1B – 3B) calculated for three ROIs of the solid metastatic foci in the lung parenchyma shown in **Fig. 3 E and J**. The calculations were performed for an interpolation factor of 16 on the second-derivative FTIR spectra.

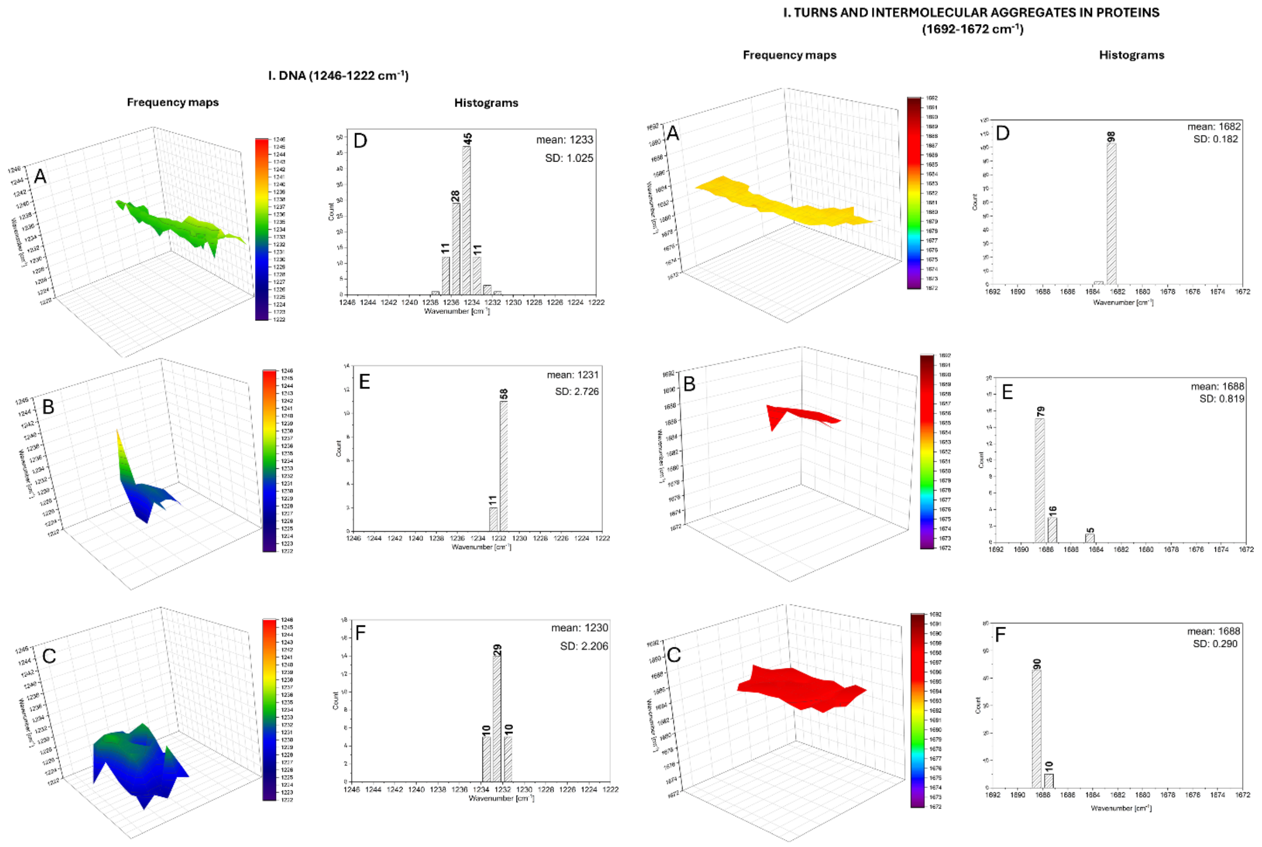

Figure S8. The comparison of the frequency maps (A-C) and their histograms (D-F) calculated for three small tumors in the lung parenchyma (19-50 pixels). The calculations were performed for an interpolation factor of 16 on the second-derivative FTIR spectra.

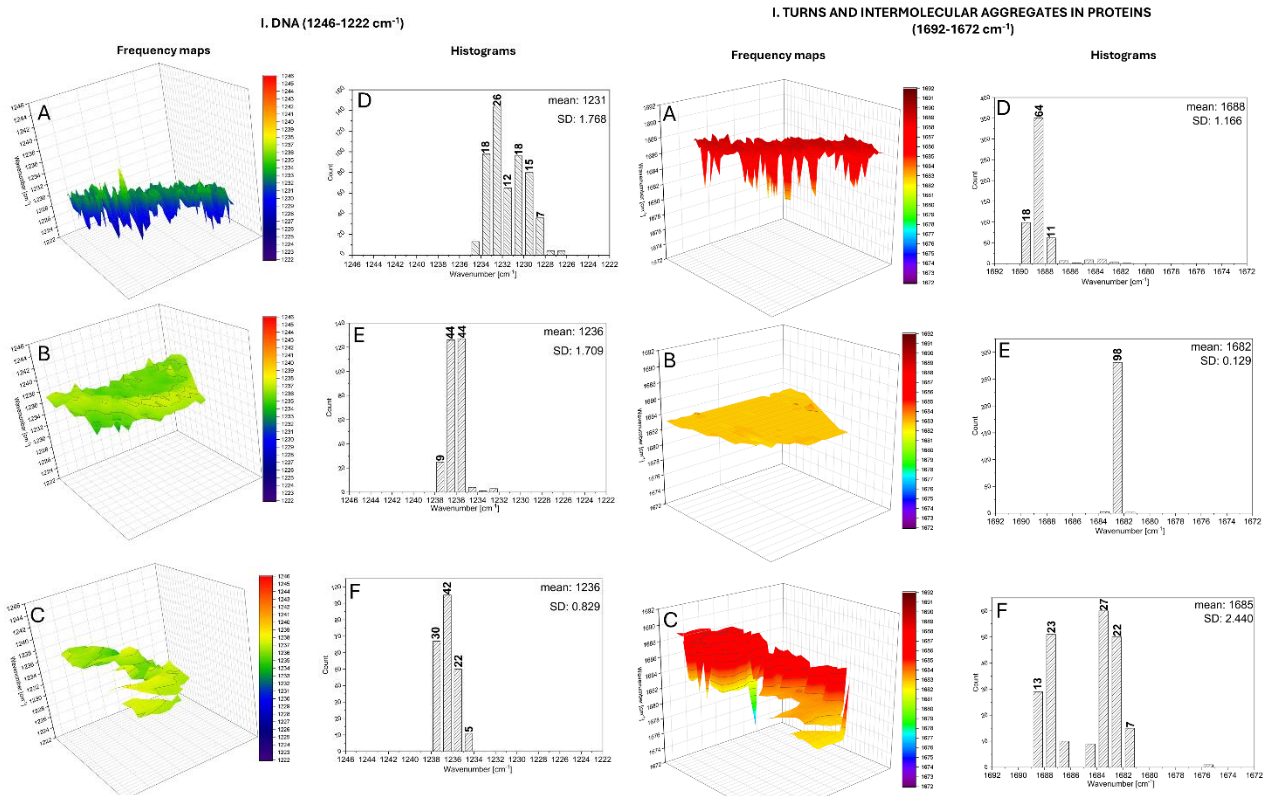

Figure S9. The comparison of the frequency maps (A-C) and their histograms (D-F) calculated for three large tumors in the lung parenchyma (400-600 pixels). The calculations were performed for an interpolation factor of 16 on the second-derivative FTIR spectra.
